# The Discrete Gaussian Expectation Maximization (Gradient) Algorithm for Differential Privacy

**DOI:** 10.1155/2021/7962489

**Published:** 2021-12-30

**Authors:** Weisan Wu

**Affiliations:** School of Mathematics and Statistics, Baicheng Normal University, Baicheng, China

## Abstract

In this paper, we give a modified gradient EM algorithm; it can protect the privacy of sensitive data by adding discrete Gaussian mechanism noise. Specifically, it makes the high-dimensional data easier to process mainly by scaling, truncating, noise multiplication, and smoothing steps on the data. Since the variance of discrete Gaussian is smaller than that of the continuous Gaussian, the difference privacy of data can be guaranteed more effectively by adding the noise of the discrete Gaussian mechanism. Finally, the standard gradient EM algorithm, clipped algorithm, and our algorithm (DG-EM) are compared with the GMM model. The experiments show that our algorithm can effectively protect high-dimensional sensitive data.

## 1. Introduction

Now, big data have spread to every field and organization in our society, generating large amounts of personal data every day, which people use and analyse to enable the rapid development of society and technology. However, it is expected that some personal private data will be protected from being hacked or made public when it is collected. Therefore, how to effectively protect the privacy of data, not to be attacked, and can be effectively used, has gradually been paid attention to. Dwork et al. [1] introduced the concept and basic theoretical framework of differential privacy, which can effectively protect users' data privacy and has a strict and elegant mathematical theoretical framework and guarantees.

Gradient EM algorithm is one of the most important statistical models, and Wang et al. [2] recently applied sensitive data for privacy protection. Before this, people used the original EM algorithm and gradient EM algorithm, and there is no statistical guarantee. Until Balakrishnan et al. [4] gave the statistical guarantee of EM algorithm, Wang et al. [3] gave the guarantee of gradient EM algorithm based on it and extended it to the data privacy protection theory. However, just like most scholars, Gaussian noise with continuous distribution is added to the data, while in practice, the data output queries are often discrete, such as the number of records in the database that meets certain conditions. For this reason, Canonne et al. [5] proposed to use a discrete Gaussian mechanism to add discrete Gaussian noise to the data and to ensure that it has the same excellent accuracy as adding continuous Gaussian noise.

In this paper, we design a discretized Gaussian algorithm based on the gradient EM algorithm for differential privacy calculation based on [2]. Our algorithm has a good practical effect and can be extended to the general standard model. Meanwhile, the corresponding statistical guarantee of the algorithm is given in this paper. The structure of this paper is as follows: in the second part, we first introduce some theories of gradient EM algorithm, discrete Gaussian, and differential privacy, as well as some works related to this paper. In the third part, we introduce our model, namely, differential privacy discrete Gaussian EM (Gradient) algorithm (DG-EM), and the relevant statistical guarantee theorem. In the fourth part, we give the data simulation of the sensitivity, sample size, and dimension of the aggregated data, and the discussion of the model and future work are shown in the fifth part. Finally, we add the proof of some lemmas in the appendix.

## 2. Preliminaries

### 2.1. Gradient EM Algorithm

Assume that (*X*, *Z*) is complete data, where *X* is an observing sample and called *Z* as a latent variable. They are generally unobservable because they are missing or have underlying data structures. We denote *𝒳* and *𝒵* as the sample space for variables *X*, *Z*, respectively. Suppose that (*X*, *Z*) has a joint density function *p*_*θ*_0__(*x*, *z*); it belongs to some parameterized distribution family {*p*_*θ*_0__*|θ*_0_ ∈ Ω}. For convenience, the variable *X* has a margin density function *π*_*θ*_(*x*)=∫_*𝒵*_*p*_*θ*_(*x*, *z*)d*z*, and *π*_*θ*_(*z|x*)=*p*_*θ*_(*x*, *z*)/*π*_*θ*_(*x*) is a *Z*′ s conditional density function which is under *X*=*x*. Suppose that the given observer samples are *x*_1_,…, *x*_*n*_ from population *X*. The EM algorithm needs to maximize the log-likelihood function *ℓ*_*n*_(*θ*)=log  *p*_*θ*_(*x*, *z*). Through Jensen's inequality, the lower bound of the log-likelihood function can be writen as follows:

(1) $$\begin{array}{c} \frac{1}{n}\left\{\ell_{n}\left(\theta \right)-\ell_{n}\left(\theta^{\prime }\right)\right\}\\ \geq \frac{1}{n}\sum_{i=1}^{n}\int_{Z}\pi_{\theta^{\prime }}\left(z|x_{i}\right)\log\;p_{\theta }\left(x_{i},z\right)dz-\frac{1}{n}\sum_{i=1}^{n}\int_{Z}\pi_{\theta^{\prime }}\left(z|x_{i}\right)\log\;p_{\theta^{\prime }}\left(x_{i},z\right)dz, \end{array}$$

where

(2) $$\begin{array}{c} q_{i}\left(\theta ,\theta^{\prime }\right)=\sum_{i=1}^{n}\int_{Z}\pi_{\theta^{\prime }}\left(z|x_{i}\right)\log\;p_{\theta }\left(x_{i},z\right)dz, \end{array}$$

(3) $$\begin{array}{c} Q_{n}\left(\theta ,\theta^{\prime }\right)=\frac{1}{n}\sum_{i=1}^{n}q_{i}\left(\theta ,\theta^{\prime }\right). \end{array}$$

The expectation of *Q*_*n*_(*θ*, *θ*′) is denoted as

(4) $$\begin{array}{c} Q\left(\theta ,\theta^{\prime }\right)=E_{x∼\pi_{\theta^{\prime }}\left(x\right)}\int_{Z}\pi_{\theta^{\prime }}\left(z|x\right)\log\;p_{\theta }\left(x,z\right)dz. \end{array}$$

To maximize equation (3), the left term of the inequality can be sufficiently large by iteratively increasing the lower bound on the right term. The standard EM algorithm [6–9] estimates the function *Q*_*n*_(*θ*, *θ*^(*t*)^) by E-step at each iteration, then the parameters are estimated in M-step to make the parameter values of this iteration maximize the function *Q*_*n*_(*θ*, *θ*^(*t*)^) and denote the parameter as *θ*^(*t*+1)^=max_*θ*∈Ω_*Q*_*n*_(*θ*, *θ*^(*t*)^). The gradient EM algorithm is usually used to achieve higher accuracy and faster global maximum if the function is differentiable at each iteration step. The gradient EM algorithm is usually stated as follows: when the function *Q*_*n*_(*θ*, *θ*^(*t*)^) is differentiable at the *t*-th iteration, we can update the current parameter *θ*^(*t*)^ to *θ*^(*t*+1)^ by the following steps:

  E-step: compute *Q*_*n*_(*θ*, *θ*^(*t*)^),
  M-step: update *θ*^(*t*+1)^=*θ*^(*t*)^+*η*∇*Q*_*n*_(*θ*^(*t*)^, *θ*^(*t*)^),

where *η* is a parameter which calls step size.

### 2.2. Discrete Gaussian

The study of discrete distributed forms of noise has received more attention this year. In the literature, people studied discrete Laplace distribution, discrete binomial distribution, and discrete Gaussian distribution and applied them to the field of cryptography.

In this paper, the differential privacy model is studied based on Gaussian mechanism. The noise with normal distribution makes the model have many elegant mathematical properties. Although the discrete Laplace noise mechanism and the discrete Gaussian noise mechanism cannot be compared in the same model, since they are used in different privacy mechanisms, we are still willing to use the discrete Gaussian noise in order to obtain aesthetic mathematical conclusions [10–13].

In this paper, we need to add noise to have discrete Gaussian distribution to specially treated sample. Firstly, we will give the definition of the discrete Gaussian distribution and some useful related theories.

**Definition 1 .**

Let *μ*, *σ*^2^ ∈ *ℝ*, *σ* > 0, if random variable *X* has probability mass function as follows:

(5) $$\begin{array}{c} \text{Pr}\left(X=x\right)=\frac{\exp\left\{-{\left(x-\mu \right)}^{2}/\left(2\sigma^{2}\right)\right\}}{\sum_{y\in \mathbb{Z}}\exp\left\{-{\left(y-\mu \right)}^{2}/\left(2\sigma^{2}\right)\right\}},\forall x\in \mathbb{Z}. \end{array}$$

On the integers support set, then we call it is a discrete Gaussian distribution with location parameter *μ* and scale parameter *σ*^2^ and denoted *N*_*ℤ*_(*μ*, *σ*^2^).

### 2.3. Some Basic Theories on Differential Privacy

In this part, we will give some basic theories on differential privacy [14, 15].

**Definition 2 .**

A randomized algorithm ℳ : *𝒳*⟶*𝒴* satisfies (*ϵ*, *δ*)-differential privacy (DP) if for all neighboring datasets ,*D*, *D*′ ⊂ *𝒳*, differing on a single entry. For all events *S* in the space *𝒴*, we have Pr(ℳ(*D*) ∈ *S*) ≤ *e*^*ϵ*^Pr(ℳ(*D*′) ∈ *S*)+*δ*. Moreover, we called its approximate differential privacy, if *δ* > 0, and we called its pure or point-wise *ϵ*-differential privacy in the case of (*ϵ*, 0)-differential privacy.

The concept of concentrated differential privacy given by Bun et al. [14] as follows:

**Definition 3 .**

A randomized algorithm ℳ : *𝒳*⟶*𝒴* satisfies *ρ*-concentrated differential privacy if for neighboring datasets *D*, *D*′ ⊂ *𝒳*, and for any *α* ∈ (1, *∞*), we have *D*_*α*_(ℳ(*D*)‖ℳ(*D*′)) ≤ *ρ*, where *D*_*α*_(*P*‖*Q*)=(1/*α* − 1)log∑_*y*_(*P*(*y*)/*Q*(*y*))^*α*^*Q*(*y*) is the Renyi divergence of order *α* of the distribution form the distribution.

From these definitions, we have the conclusion that pure-DP can imply *ρ*-CDP, and *ρ*-CDP can imply $\left(\rho +2\sqrt{\rho \;\log\;\delta^{-1}},\delta \right)$-DP, where *δ* is a positive constant.

In order to ensure the consistency of the parameters of our model, we need some basic definitions and assumptions based on [4].

**Definition 4 .**

(self-consistent). We called the function *Q*(·; *θ*^*∗*^) is self-consistent if *θ*^*∗*^=argmax_*θ*∈Ω_*Q*(*θ*; *θ*^*∗*^).

**Definition 5 .**

(Lipschitz-gradient-2 (*L*, ℬ)). We called the function *Q*(·; ·) is Lipschitz-gradient-2 (*L*, ℬ), if we have the following inequality for parameter *θ*^*∗*^ and *θ* ∈ ℬ:

(6) $$\begin{array}{c} {\left\|\nabla Q_{n}\left(\theta ;\theta^{*}\right)-\nabla Q_{n}\left(\theta ;\theta \right)\right\|}_{2}\leq L{\left\|\theta -\theta^{*}\right\|}_{2}. \end{array}$$

**Definition 6 .**

(*μ*-smooth). We call the function *Q*(·; ·) is *μ*-smooth, if for any parameters *θ*, *θ*′ ∈ ℬ, we have the inequality

(7) $$\begin{array}{c} Q\left(\theta ;\theta^{\prime }\right)\geq Q\left(\theta^{\prime };\theta^{*}\right)+{\left(\theta -\theta^{\prime }\right)}^{T}\nabla Q\left(\theta^{\prime };\theta^{*}\right)-\frac{\mu }{2}{\left\|\theta -\theta^{\prime }\right\|}_{2}^{2}. \end{array}$$

**Definition 7 .**

(*λ*-strongly concave). We call the function *Q*(·; *θ*^*∗*^) is *λ*-strongly concave, if for any parameters *θ*, *θ*′ ∈ ℬ, we have the inequality

(8) $$\begin{array}{c} Q\left(\theta ;\theta^{\prime }\right)\leq Q\left(\theta^{\prime };\theta^{*}\right)+{\left(\theta -\theta^{\prime }\right)}^{T}\nabla Q\left(\theta^{\prime };\theta^{*}\right)-\frac{\lambda }{2}{\left\|\theta -\theta^{\prime }\right\|}_{2}^{2}. \end{array}$$

**Assumption 1 .**

We assume that the function *Q*(·; ·) is self-consistent, Lipschitz-gradient-2 (*L*, ℬ), *μ*-smooth, and *λ*-strongly concave on some parameter sets ℬ.

## 3. Differential Privacy Discrete Gaussian EM (Gradient) Model

We will mention that the EM algorithm based on [2] and use the discrete Gaussian noise mechanism of high-dimensional truncation algorithm, which satisfies the centralized differential privacy (CDP). Like Wang et al. [2], we have first considered one coordinate case that is 1-dimensional random variable *x*. Let *x*_1_,…, *x*_*n*_ be i.i.d. sampled from *x*. We get the clipped estimator as follows:

*Step 1.* For the sample *x*_*i*_, we take a soft truncation function *h*(*x*) which is defined by Catoni and Giulini [16],
  (9) $$\begin{array}{c} h\left(x\right)=\left\{\begin{array}{c} -\frac{2\sqrt{2}}{3},x<-\sqrt{2}\\ x-\frac{x^{3}}{6},-\sqrt{2}\leq x\leq \sqrt{2}\\ \frac{2\sqrt{2}}{3},x>\sqrt{2} \end{array}\right.. \end{array}$$
Then, we take some mild constant *ω* and rescaled sample *x*_*i*_ by dividing *ω* to get *h*(*x*_*i*_/*ω*); through this approach, we can get the truncated mean as follows:
  (10) $$\begin{array}{c} \frac{\omega }{n}\sum_{i=1}^{n}h\left(\frac{x_{i}}{\omega }\right)\approx E\left(X\right). \end{array}$$
From the expression of the function *h*(*x*), we know *h*(*x*) is bounded by $\left(2\sqrt{2}/3\right)$, so the sensitivity is $\left(4\sqrt{2}/3\right)$.
*Step 2.* Generate random noises *o*_1_,…, *o*_*n*_ from a common distribution *o* ~ *χ* with *𝔼*(*o*)=0. For data *x*_*i*_, we get a new data *x*_*i*_(1+*o*_*i*_) though multiply the noise factor 1+*o*_*i*_, and we get term *h*(*x*_*i*_(1+*o*_*i*_)/*ω*) by scaling and truncation step. Finally, we get
  (11) $$\begin{array}{c} \tilde{x}\left(o\right)=\frac{\omega }{n}\sum_{i=1}^{n}h\left(\frac{x_{i}\left(1+o_{i}\right)}{\omega }\right). \end{array}$$
Multiplicative noise is an effective method to ensure the estimation effect of typical points and increase the estimation effect of outliers as much as possible. It was first proposed by Srivastava et al. [17], and the motivation of using Gaussian multiplicative noise comes from [18].
*Step 3.* Finally, we take the expectation for the distributions with arrive multiplicative noise as follows:
  (12) $$\begin{array}{c} \hat{x}=E\left(\tilde{x}\left(o\right)\right)=\frac{\omega }{n}\sum_{i=1}^{n}\int h\left(\frac{x_{i}\left(1+o_{i}\right)}{\omega }\right)d\chi \left(o_{i}\right). \end{array}$$

Like Catoni and Giulini [16], taking *χ* ~ *N*(0, (1/*β*)), we take the distribution *χ* following the discrete Gaussian distribution as *χ* ~ *N*_*ℤ*_(0, (1/*β*)). Easily, for any given constant *a*, *b* > 0, we also have

(13) $$\begin{array}{c} E_{\chi }\left(h\left(a+b\sqrt{\beta }o\right)\right)=a\left(1-\frac{b^{2}}{2}\right)-\frac{a^{3}}{6}+R\left(a,b\right), \end{array}$$

where *R*(*a*, *b*) is a correction term *R*(*a*, *b*)=*T*_1_+*T*_2_+*T*_3_+*T*_4_+*T*_5_. Signs *T*_1_ − *T*_5_ are respectively denoted as

(14) $$\begin{array}{c} T_{1}=\frac{2\sqrt{2}}{3}\left(F_{-}-F_{+}\right),\\ T_{2}=-\left(a-\frac{a^{3}}{6}\right)\left(F_{-}+F_{+}\right),\\ T_{3}=\frac{b}{\sqrt{2\pi }}\left(1-\frac{a^{2}}{2}\right)\left(E_{-}-E_{+}\right),\\ T_{4}=\frac{ab^{2}}{2}\left(F_{-}+F_{+}+\frac{1}{\sqrt{2\pi }}\left(V_{+}E_{+}-V_{-}E_{-}\right)\right),\\ T_{5}=\frac{b^{3}}{6\sqrt{2\pi }}\left(\left(2+V_{-}^{2}\right)E_{-}-\left(2+V_{+}^{2}\right)E_{+}\right). \end{array}$$

Also, the notation is defined by

(15) $$\begin{array}{c} V_{-}=\frac{\sqrt{2}-a}{b},\\ V_{+}=\frac{a+\sqrt{2}}{b},\\ E_{-}=\exp\left(-\frac{V_{-}^{2}}{2}\right),\\ E_{+}=\exp\left(-\frac{V_{+}^{2}}{2}\right),\\ F_{-}=\Phi \left(-V_{-}\right),\\ F_{+}=\Phi \left(-V_{+}\right). \end{array}$$

Unproved, we have the following estimation error Lemma 1 which is like Lemma 5 in Holland [19], and we gave the proof of it in Appendix A.

**Lemma 1 .**

Let *x*_1_,…, *x*_*n*_ be i.i.d. sampled form *x* ~ *μ*. Assume *𝔼*_*μ*_*x*^2^ ≤ *τ*, and the upper bound has known. Given a number 0 < *γ* < 1, for *β*=2  log(*γ*^−1^) and $\omega =\sqrt{\left(n\tau /2\;\log\left(\gamma^{-1}\right)\right)}$, we have

(16) $$\begin{array}{c} \left|\hat{x}-E_{\mu }\left(x\right)\right|\leq O\left(\sqrt{\frac{\tau \;\log\left(\gamma^{-1}\right)}{n}}\right), \end{array}$$

with probability at least 1 − *γ*.

From the soft truncation function and the multiplicative noise algorithm, we know that the sensitivity of the processed observation samples is $\left(4\sqrt{2}s/3n\right)$. Next, we need to add discrete Gaussian noise to the observations and obtain that the query

(17) $$\begin{array}{c} ℳ\left(D\right)=\hat{x}+Y,Y∼N_{\mathbb{Z}}\left(0,\sigma^{2}\right),\sigma^{2}=O\left(\frac{\omega^{2}\log\left(\delta^{-1}\right)}{\epsilon^{2}n^{2}}\right), \end{array}$$

will be (*ϵ*, *δ*)-DP, which leads the following Lemma 3; we give the proof in Appendix B.

**Lemma 2 .**

Let *ϵ* > 0; let the function *q* : *𝒳*^*n*^⟶*ℤ* be an operator algorithm which is defined by Steps 1–3, satisfying |*q*(*x*) − *q*(*x*′)| ≤ Δ for any *x*, *x*′ ∈ *𝒳*^*n*^; the query can be writen as randomized algorithm ℳ : *𝒳*^*n*^⟶*ℤ* by ℳ(*D*)=*q*(*x*)+*Y*, where *Y* ~ *N*_*ℤ*_(0, *σ*^2^), then ℳ satisfies (*ϵ*, *δ*)-DP.

Furthermore, these results imply the following lemma.

**Lemma 3 .**

Under the assumptions in Assumption 1, with probability at least 1 − *γ*, the following holds:

(18) $$\begin{array}{c} \left|ℳ\left(D\right)-E\left(x\right)\right|\leq O\left(\frac{\Delta \;\log\left(\delta^{-1}\right)}{\epsilon^{2}}\right). \end{array}$$

After the estimation of the univariate private data, in the *t*-th iteration of Algorithm 1, we use the univariate estimation method for each coordinate of the gradient ∇*Q*_*n*_(*θ*^(*t*)^; *θ*^(*t*)^) and then get the estimation of the gradient ∇*Q*_*n*_(*θ*^(*t*)^; *θ*^(*t*)^). Finally, step M is performed.

**Lemma 4 .**

For any 0 < *ϵ* < 1, let *D*_*α*_(ℳ(*x*)‖ℳ(*x*′)) ≤ *ϖ*; for any *α* ∈ (1, *∞*), *ϵ* ≥ 0, and *x*, *x*′ ∈ *𝒳*^*n*^, Algorithm 1 satisfies (*ϵ*, *δ*)-DP for

(19) $$\begin{array}{c} \delta =\frac{\exp\left(\left(\alpha -1\right)\left(\varpi -\epsilon \right)\right)}{\alpha -1}{\left(1-\frac{1}{\alpha }\right)}^{\alpha }, \end{array}$$

where *Y* ~ *N*_*ℤ*_(0, *σ*^2^).

For Algorithm 1, the next theorem shows that the parameter estimation is consistent if the initial parameter *θ*^*Init*^ is close to the true parameter *θ*^*∗*^ enough. After some simple calculations, we conclude that in Lemma 2, the upper bound is Δ=(*nτ*+*ω*_*op*_^2^/*nω*_*op*_){1+[(1/4)log(3*nτ*/2*ω*_*op*_^2^)+log(*γ*^−1^)]^−1^}, where *ω*_*op*_ is the optimal numerical solution to the equation

(20) $$\begin{array}{c} 2\omega^{2}+nE_{\mu }\left(x^{2}\right)=\omega^{2}\log\left(\frac{3nE_{\mu }\left(x^{2}\right)}{2\omega^{2}}\gamma^{-2}\right). \end{array}$$

**Lemma 5 .**

Let ℬ={*θ* : ‖*θ* − *θ*^*∗*^‖_2_ ≤ *R*} denote a parameter set with *R*=*κ*‖*θ*^*∗*^‖_2_^2^, *κ* ∈ (0,1) which is a positive constant. Assume parameters *L*, ℬ, *μ*, *λ*, *τ* satisfying condition of 1 − 2(*λ* − *L*/*λ*+*μ*) ∈ (0,1). If ‖*θ*^*Init*^ − *θ*^*∗*^‖_2_ ≤ *R*/2 and *n* is a large number such that

(21) $$\begin{array}{c} \tilde{\Omega }\left({\left(\frac{1}{\lambda -L}\right)}^{2}\frac{d^{2}T\tau \;\log\left(\gamma^{-1}\right)}{\epsilon^{2}R^{2}}\right)\leq n. \end{array}$$

We have Pr(*θ*^(*t*)^ ∈ ℬ) ≥ 1 − 2*Tγ* for all *t* ∈ [*T*]. Furthermore, if we take *T*=*O*((*λ*+*μ*/*λ* − *L*)log(*n*)) and *η*=(2/*λ*+*μ*), we have

(22) $$\begin{array}{c} {\left\|\theta^{\left(T\right)}-\theta^{*}\right\|}_{2}\leq \tilde{O}\left(R\sqrt{\frac{\lambda +\mu }{{\left(\lambda -L\right)}^{3}}}\frac{d\;\log\left(\delta^{-1}\right)\log\left(\gamma^{-1}\sqrt{\tau }\right)}{\sqrt{n\epsilon^{2}}}\right). \end{array}$$

**Lemma 6 .**

Let (‖*θ*^*∗*^‖/*σ*) ≥ *r*, then there exists a constant *C* such that the properties of self-consistent Lipschitz-gradient-2(*L*, ℬ), *μ*-smoothness, and *λ*-strongly concave hold for the function *Q*(·; ·) with *L*=exp(−*Cr*), *μ*=*λ*=1, *R*=*κ*‖*θ*^*∗*^‖_2_, *κ*=1/4, ℬ={*θ* : ‖*θ* − *θ*^*∗*^‖ ≤ *R*}, where *r* is a enough large constant means that the minimum signal-to-noise ratio (SNR).

Furthermore, we can get Theorems 1 and 2. The proof of these theorems is very simple; we do not list the detailed proof procedure here. In fact, we only need to replace the upper bound on the variance of the discrete noise in [2] with a single coordinate with 3  exp(−1/2*σ*^2^).

**Theorem 1 .**

With the same condition as in Lemma 4, for any *θ* ∈ ℬ, the *j*-th coordinate of ∇*q*(*θ*; *θ*) satisfies the following results:

(23) $$\begin{array}{c} E_{y}{\left(\nabla_{j}q\left(\theta ;\theta \right)\right)}^{2}\leq O\left({\left\|\theta^{*}\right\|}_{\infty }^{2}+3\;\exp\left(-\frac{1}{2\sigma^{2}}\right)\right). \end{array}$$

**Theorem 2 .**

With the same conditions in Lemma 3, we assume that ‖*θ*^*Init*^ − *θ*^*∗*^‖_2_ ≤ (‖*θ*^*∗*^‖_2_^2^/8) in Algorithm 1, and *n* is a large enough number such that

(24) $$\begin{array}{c} \tilde{\Omega }\left(\frac{n\left({\left\|\theta^{*}\right\|}_{\infty }^{2}+3\;\exp\left(-\left(1/2\sigma^{2}\right)\right)\right)+\omega_{op}^{2}}{\omega_{op}\epsilon^{2}{\left\|\theta^{*}\right\|}_{2}^{2}}d^{2}\left\{1+{\left[\frac{1}{4}\log\left(\frac{3n\tau }{2\omega_{op}^{2}}\right)+\log\left(\gamma^{-1}\right)\right]}^{-1}\right\}\right)\leq n. \end{array}$$

If we take *T*=*O*(log(*n*)) and the ratio as *η*=*O*(1), then for a failure probability *γ*, we have with probability at least 1 − 2*Tγ*

(25) $$\begin{array}{c} {\left\|\theta^{\left(T\right)}-\theta^{*}\right\|}_{2}\leq \tilde{O}\left({\left\|\theta^{*}\right\|}_{2}\frac{n\sqrt{{\left\|\theta^{*}\right\|}_{\infty }^{2}+3\;\exp\left(-\left(1/2\sigma^{2}\right)\right)}+\omega_{op}^{2}}{\sqrt{n\epsilon^{2}}\omega_{op}}d\left\{1\right.\right.\\ \left.\left.+{\left[\frac{1}{4}\log\left(\frac{3n\tau }{2\omega_{op}^{2}}\right)+\log\left(\gamma^{-1}\right)\right]}^{-1}\right\}\right). \end{array}$$

We note that Lemmas 3–6 and Theorems 1 and 2 are easy to get through Lemmas 1 and 2. Due to limited space, we delete these proofs here, and readers can prove them by themselves. It is only necessary to pay attention to the upper bound of the *ℓ*_2_-norm between the iterative values of parameters and the truth values in the process of proof.

## 4. Experiments and Results

In this section, we will evaluate the performance of Algorithm 1 on the GMM model based on these methods. We will study the statistical setting and theoretical behavior of this algorithm on synthetic data.

### 4.1. Baseline Methods

In this part, we will compare the two methods primarily. For convenience, we will refer to the gradient EM algorithm as EM, which will serve as a nonprivate baseline method. The other is the clipped differential private EM algorithm, which we still refer to as clipped [20], which will serve as our privacy baseline approach.

### 4.2. Experimental Settings

In this experiment, we generate the synthetic data of the mixed distribution of two components. To generate each of the algorithm, we consider the random initialization method for the selection of initial parameter values. In the results, we used to measure the resulting estimation error. We set signal-to-noise ratio (‖*θ*^*∗*^‖/*σ*)=3. For the privacy parameter *ϵ*, we set *ϵ*={0.5, 0.8, 1}, and then the parameter *δ*=Pr(*Y* > (*ϵσ*^2^/Δ)+(Δ/2)) needs to calculate because it is the function of *ϵ*.

### 4.3. Experimental Results

**Figure 1:**
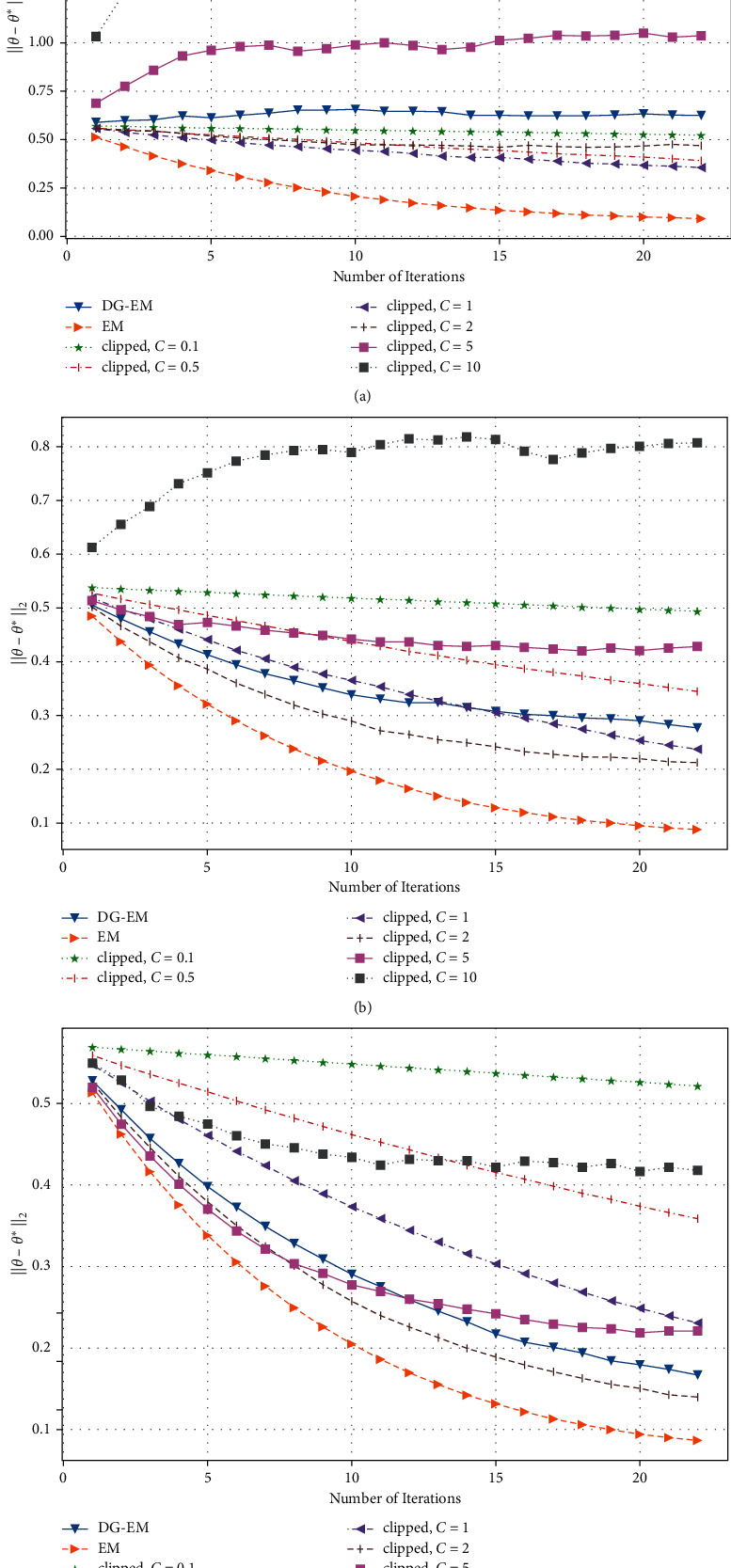
Estimation error of GMM clipped vs. iteration *t* under different clipping threshold *C* and budgets *ϵ*. (a) *n* = 1000; *d* = 20; *ϵ*  = 0.2, (b) *n* = 1000; *d* = 20; *ϵ* = 0.5, and (c) *n* = 1000; *d* = 20; *ϵ* = 1.

As can be seen from Figure 1, we fixed *n*=1000, *d*=20. When the budget of our method is set at different values, the estimation error decreases significantly with the increase of iteration time. When the budget is 0.2, 0.5, and 1, the optimal value is 1,2, and 2, respectively. It is difficult for us to determine the optimal value *C*.

**Figure 2:**
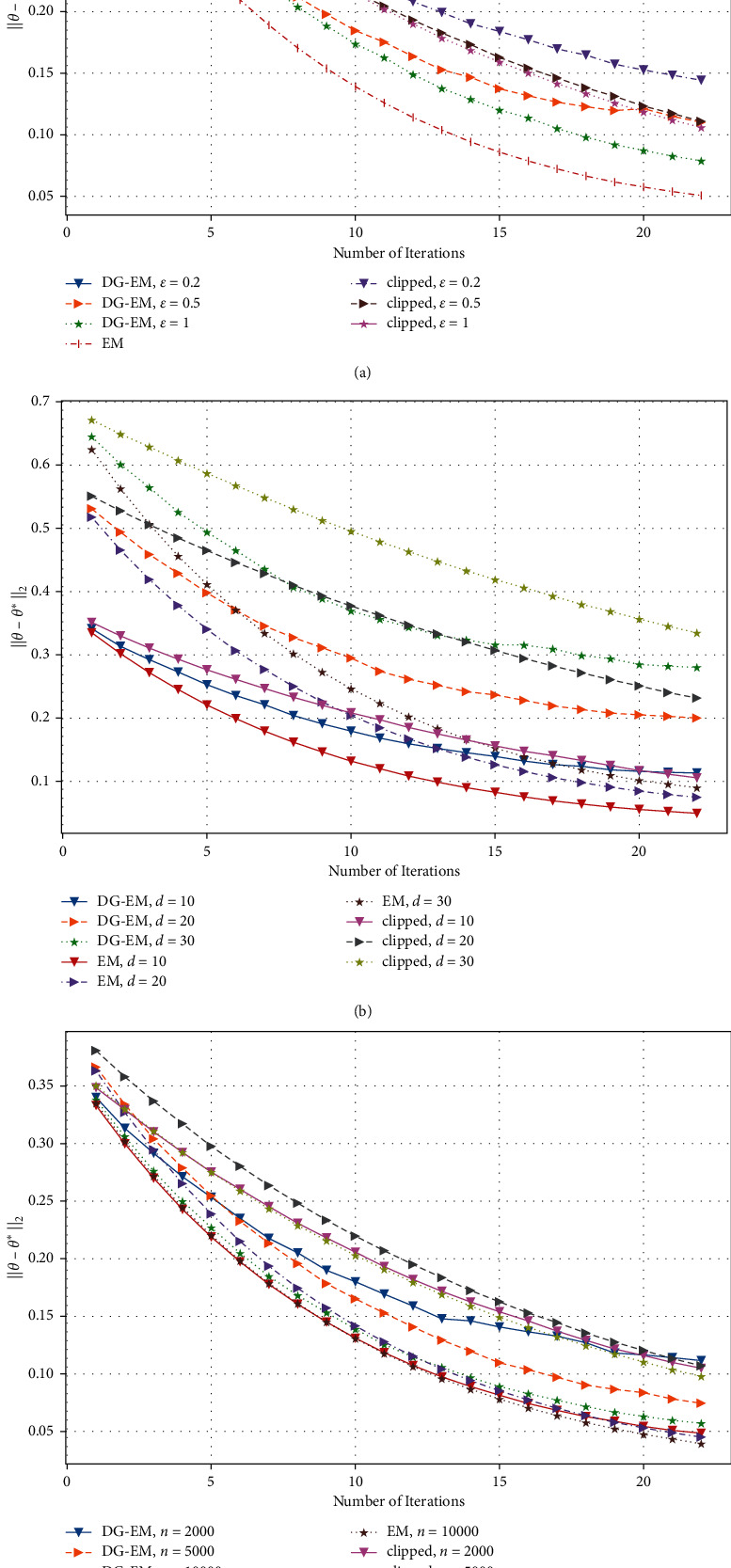
Estimation error of GMM w.r.t. privacy budget *ϵ*, data dimension (lower) *d*, data size *n*, and iteration *t*. (a) *n* = 2000; *d* = 10, (b) *n* = 2000; *ϵ*  = 0.5, and (c) *d* = 10, *ϵ* = 0.5.

In Figure 2, under the lower dimension case, we test how the data dimension *d*, privacy budget *ϵ*, and data size *n* affect the estimation error ‖*θ* − *θ*^*∗*^‖_2_ of algorithms on the Gaussian mixture model over iteration *t*. We can see that the estimation error of Algorithm 1 in GMM decreases when *ϵ* increases, *n* increases, or *d* decreases. However, we can see that when the budget *ϵ* is small, the effect of our algorithm is performed badly, and the estimation error declines unstably with the increase of the number of iterations.

**Figure 3:**
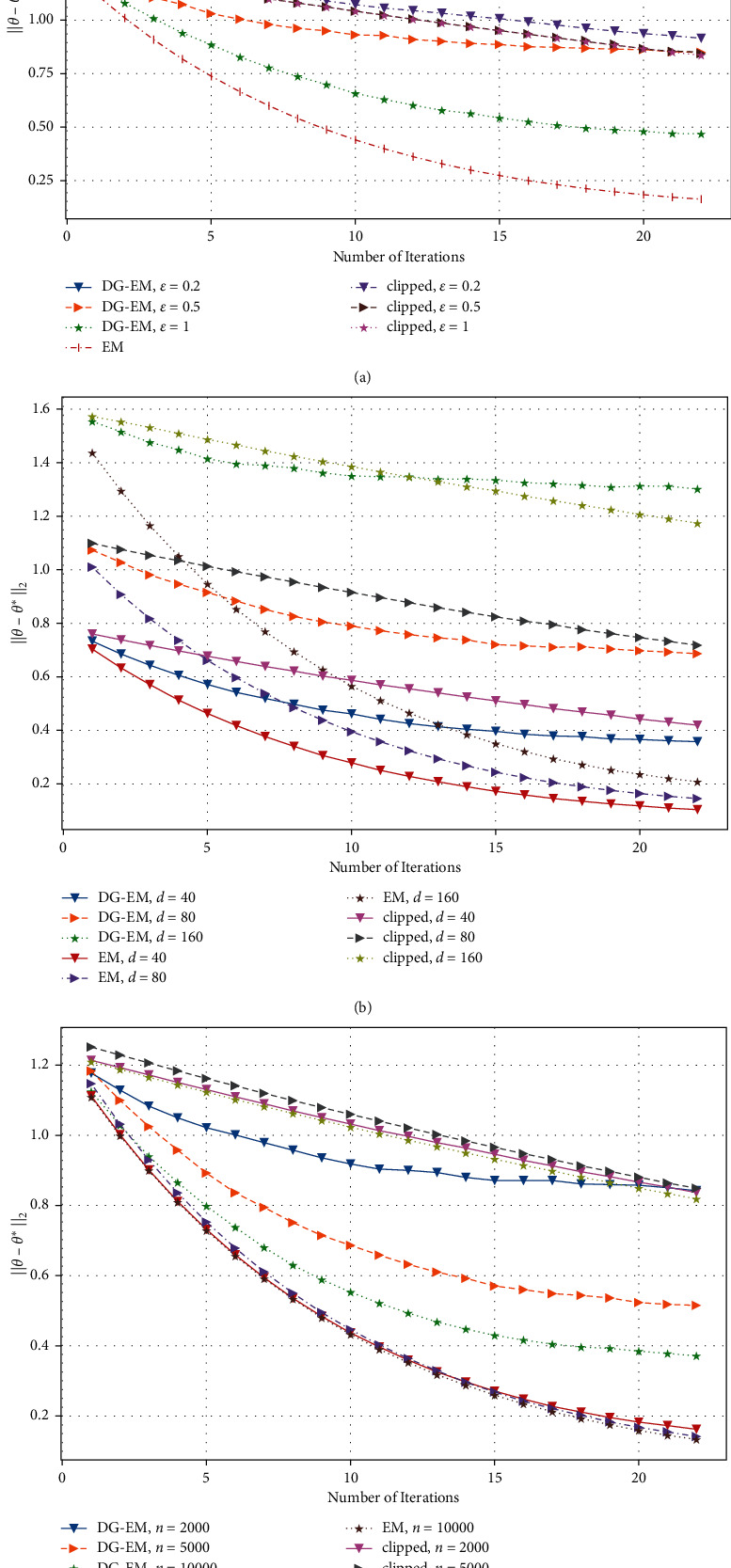
Estimation error of GMM w.r.t. privacy budget *ϵ*, data dimension (higher) *d*, data size *n*, and iteration *t*. (a) *n* = 2000; *d* = 100, (b) *n* = 2000; *ϵ* = 0.5, and (c) *d* = 100, *ϵ* = 0.5.

In Figure 3, we can see that, in the face of high-dimensional data, the effect of estimation error ‖*θ* − *θ*^*∗*^‖_2_ needs a relatively large sample to be guaranteed. We conducted experiments with higher dimensions *d*=40,80,160 and different sample sizes of 2000,5000, and 10 000, respectively. It can be seen that when the sample size *n* is large enough, the estimation error can be guaranteed to decrease significantly with the number of iterations *t*. As shown in Figure 3, with the increase of sample size, our algorithm is equally effective in high-dimensional space, which is not comparable with Wang et al.'s [2] algorithm.

## 5. Conclusions

In this paper, we study the differential privacy model with discrete Gaussian mechanism noise. Through the process of data scaling and truncation, the model effectively solves the influence of high-dimensional data on the model. Through the experimental part and theoretical proof, we can see that the estimation error of the model adding discrete Gaussian noise is faster than that of the model adding continuous Gaussian noise in the low dimension than that of the clipped model. The effect is much better than that of [2] in the case of high dimension. At the same time, in the previous lemma section, we can see that our model has more compact bounds, because of the smaller variance of discrete Gaussian noise.

## Figures and Tables

**Algorithm 1:**
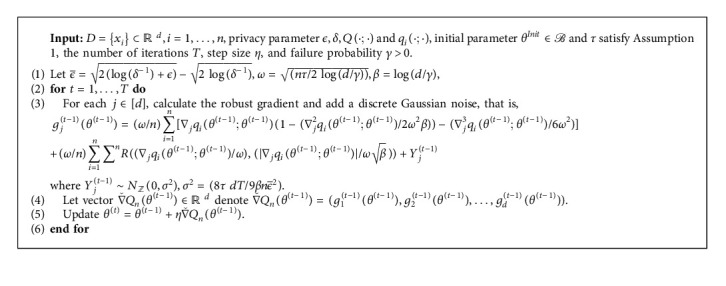
Differentially private DG-EM (gradient) algorithm.

## Data Availability

The data in this paper are random numbers generated by statistical software R.

## Appendix

### A

Proof of Lemma 1

Proof In order to make the conclusion universal, we make some necessary assumptions. Firstly, let *𝒫*(*ℝ*) denote all probability measures on *ℝ*, and we assumed it has an appropriate *σ*-field. Consider any two measures *v*, *v*′ ∈ *𝒫*(*ℝ*), and *f*_0_ : *ℝ*⟶*ℝ* is a *v*′-measurable function. We take the form of a cumulative generating function as

(A.1) $$\begin{array}{c} \underset{v}{\text{sup}}\left(\int f_{0}\left(u\right)dv\left(u\right)-D_{\alpha }\left(v\left\|v^{\prime }\right.\right)\right)=\log\left(\int \exp\left(f_{0}\left(u\right)\right)dv^{\prime }\left(u\right)\right), \end{array}$$

through a Legendre transform of the mapping *v*⟶*D*_*α*_(*v*‖*v*′) like [16], where *D*_*α*_(*v*‖*v*′) denotes the Renyi divergence between *v* and *v*′. Because *h*(*x*_*i*_(1+*o*_*i*_)/*ω*), *i* ∈ [*n*] is depend on two random quantities *x*_*i*_ and the noise *o*_*i*_, we write *f*(*o*, *x*)≜*h*(*x*(1+*o*)/*ω*), *o*, *x* ∈ *ℝ*. By the definition of function *h*(·) before, the function *f* : *ℝ*⟶*ℝ* is measurable and bounded with $\left(2\sqrt{2}/3\right)$. Next, we let

(A.2) $$\begin{array}{c} f_{0}\left(o\right)=\sum_{i=1}^{n}f\left(o,x_{i}\right)-c\left(o\right), \end{array}$$

where *c*(*o*) is a term needs to be determined later. Inserting *f*_0_(*o*) to (A.1), we have

(A.3) $$\begin{array}{c} B\;≜\underset{v}{\text{sup}}\left(\int f_{0}\left(o\right)dv\left(o\right)-D_{\alpha }\left(v\left\|v^{\prime }\right.\right)\right)\\ =\log\left(\int \exp\left(\sum_{i=1}^{n}f\left(o,x_{i}\right)-c\left(o\right)\right)dv\left(o\right)\right). \end{array}$$

Furthermore, we have

(A.4) $$\begin{array}{c} E_{\mu }\left(\exp\left(B\right)\right)=E_{\mu }\left(\int \frac{\exp\left(\sum_{i=1}^{n}f\left(o,x_{i}\right)\right)}{\exp\left(c\left(o\right)\right)dv\left(o\right)}\right)\\ =\int \prod_{i=1}^{n}E_{\mu }\frac{\exp\left.\left(f\left(o,x_{i}\right)\right)\right)}{\exp\left(c\left(o\right)\right)dv\left(o\right)}. \end{array}$$

If

(A.5) $$\begin{array}{c} c\left(o\right)=n\;\log\left(E_{\mu }\exp\left(f\left(o,x\right)\right)\right), \end{array}$$

we have

(A.6) $$\begin{array}{c} E_{\mu }\left(\exp\left(B\right)\right)=\int \left(\prod_{i=1}^{n}\frac{E_{\mu }\exp\left(f\left(o,x_{i}\right)\right)}{{\left[E_{\mu }\exp\left(f\left(o,x\right)\right)\right]}^{n}}\right)dv\left(o\right)=1. \end{array}$$

So,

(A.7) $$\begin{array}{c} \text{Pr}\left(B\geq \;\log\left(\gamma^{-1}\right)\right)=\text{Pr}\left(\exp\left(B\right)\geq \gamma^{-1}\right)\\ =E_{\mu }I\left\{\gamma \;\exp\left(B\right)\geq 1\right\}\\ \leq E_{\mu }\left(\gamma \;\exp\left(B\right)\right)\\ =\gamma . \end{array}$$

Because *c*(*o*) is *v*-measurable, the *f*_0_(*o*) is *v*-measurable. We have

(A.8) $$\begin{array}{c} \underset{v}{\text{sup}}\left(\int f_{0}\left(o\right)dv\left(o\right)-D_{\alpha }\left(v\left\|v^{\prime }\right.\right)\right)=\log\left(\gamma^{-1}\right), \end{array}$$

with probability at least 1 − *γ*. We have the following inequality:

(A.9) $$\begin{array}{c} \frac{1}{n}\sum_{i=1}^{n}\int f\left(o,x_{i}\right)dv\left(o\right)\leq \int \log\left(E_{\mu }\exp\left(f\left(o,x\right)\right)\right)dv\left(o\right)+\frac{D_{\alpha }\left(v\left\|v^{\prime }\right.\right)+\log\left(\gamma^{-1}\right)}{n}. \end{array}$$

Since the noise terms *o*_1_,…, *o*_*n*_ are independent and follow distribution *o* ~ *v*, we can get

(A.10) $$\begin{array}{c} \hat{x}=\frac{s}{n}\sum_{i=1}^{n}\int h\left(\frac{x_{i}\left(1+o_{i}\right)}{\omega }\right)dv\left(o_{i}\right)\\ =\frac{s}{n}\sum_{i=1}^{n}\int f\left(o,x_{i}\right)dv\left(o_{i}\right). \end{array}$$

Thus, we have the bound from equation (A.9) as follows:

(A.11) $$\begin{array}{c} \hat{x}\leq s\int \log\left(E_{\mu }\exp\left(h\left(\frac{x\left(1+o\right)}{\omega }\right)\right)\right)dv\left(o\right)+\frac{s}{n}\left[D_{\alpha }\left(v\left\|v^{\prime }\right.\right)+\log\left(\gamma^{-1}\right)\right], \end{array}$$

and then we need to analyse the first term and the second term on the right-hand side of the top inequality (A.11). For the first term, from the definition of the truncation function *h*(·), by (A.11), we have

(A.12) $$\begin{array}{c} \int \log\left(E_{\mu }\exp\left(h\left(\frac{x\left(1+o\right)}{\omega }\right)\right)\right)dv\left(o\right)\leq \int \log\left[1+\frac{\left(1+o\right)E_{\mu }\left(x\right)}{s}+\frac{{\left(1+o\right)}^{2}E_{\mu }\left(x^{2}\right)}{s^{2}}\right]dv\left(o\right)\\ \leq \int \log\left[\frac{\left(1+o\right)E_{\mu }\left(x\right)}{s}+\frac{{\left(1+o\right)}^{2}E_{\mu }\left(x^{2}\right)}{s^{2}}\right]dv\left(o\right)\\ =\frac{E_{v}\left(1+o\right)E_{\mu }\left(x\right)}{s}+\frac{E_{v}{\left(1+o\right)}^{2}E_{\mu }\left(x^{2}\right)}{s^{2}}\\ =\frac{E_{\mu }\left(x\right)}{s}+\frac{E_{\mu }\left(x^{2}\right)}{s^{2}}\left(1+\frac{1}{\theta }\right). \end{array}$$

Since *o* ~ *v*=*N*(0, (1/*θ*)), the expectation and variance of the 1+*o* are as follows:

(A.13) $$\begin{array}{c} E_{v}\left(1+o\right)=1,E_{v}{\left(1+o\right)}^{2}=\frac{1}{\theta }+{\left[E_{v}\left(1+o\right)\right]}^{2}=\frac{1}{\theta }+1. \end{array}$$

For the second term in (A.11), we need to evaluate *D*_*α*_(*v*‖*v*′). We take *v*′=*N*(0, (1/*θ*)); through simple computations, we can get

(A.14) $$\begin{array}{c} D_{\alpha }\left(v\left\|v^{\prime }\right.\right)==\frac{1}{\alpha -1}\log\left(\int {\left(dv\right)}^{\alpha }{\left(dv^{\prime }\right)}^{1-\alpha }du\right)\\ =\frac{{\left(1-0\right)}^{2}}{2\theta^{-1}}\alpha \\ =\frac{\theta \alpha }{2}. \end{array}$$

Thus, we can take the upper bound form as

(A.15) $$\begin{array}{c} \hat{x}\leq E_{\mu }\left(x\right)+\frac{E_{\mu }\left(x^{2}\right)}{2s}\left(1+\frac{1}{\theta }\right)+\frac{s}{n}\left(\frac{\theta \alpha }{2}+\log\left(\gamma^{-1}\right)\right). \end{array}$$

We take the differential for the variable *s*; we have

(A.16) $$\begin{array}{c} s^{2}=\left(1+\frac{1}{\theta }\right)E_{\mu }\left(x^{2}\right){\left(\frac{\theta \alpha }{2}+\log\left(\gamma^{-1}\right)\right)}^{-1}, \end{array}$$

and with respect to *θ*, we have

(A.17) $$\begin{array}{c} \theta^{2}=\frac{nE_{\mu }\left(x^{2}\right)}{\alpha s^{2}}. \end{array}$$

Plugging equation (A.17) into the setting of *s*, we can get

(A.18) $$\begin{array}{c} s^{2}=\frac{nE_{\mu }\left(x^{2}\right)}{2\;\log\left(\gamma^{-1}\right)}. \end{array}$$

We can get the setting of *s* from equation (A.18); equation (A.15) has upper bound with form as follows:

(A.19) $$\begin{array}{c} \hat{x}\leq E_{\mu }\left(x\right)+\sqrt{\frac{2E_{\mu }\left(x^{2}\right)\log\left(\gamma^{-1}\right)}{n}}+\sqrt{\frac{E_{\mu }\left(x^{2}\right)}{n}}. \end{array}$$

To get lower bounds on $\hat{x}-{𝔼}_{\mu }\left(x\right)$, we need to get the upper bounds on $-\hat{x}-{𝔼}_{\mu }\left(x\right)$. Similar to the analysis above, we get the upper bound of $-\hat{x}$ through

(A.20) $$\begin{array}{c} -\hat{x}\leq s\int \log\left(E_{\mu }\exp\left(-h\left(\frac{x\left(1+o\right)}{\omega }\right)\right)dv\left(o\right)+\frac{s}{n}\left[D_{\alpha }\left(v\left\|v^{\prime }\right.\right)+\log\left(\gamma^{-1}\right)\right]\right.. \end{array}$$

By the fact

(A.21) $$\begin{array}{c} -\log\left(\frac{1-x+x^{2}}{2}\right)\leq \psi \left(x\right)\leq \;\log\left(\frac{1-x+x^{2}}{2}\right), \end{array}$$

we have

(A.22) $$\begin{array}{c} \int \log\left(E_{\mu }\exp\left(-h\left(\frac{x\left(1+o\right)}{\omega }\right)\right)dv\left(o\right)\right. \end{array}$$

(A.23) $$\begin{array}{c} \hat{x}\leq -E_{\mu }\left(x\right)+\sqrt{\frac{2E_{\mu }\left(x^{2}\right)\log\left(\gamma^{-1}\right)}{n}}+\sqrt{\frac{E_{\mu }\left(x^{2}\right)}{n}}. \end{array}$$

Putting the above analysis together, we have

(A.24) $$\begin{array}{c} \hat{x}-E_{\mu }\left(x\right)\geq \sqrt{\frac{2E_{\mu }\left(x^{2}\right)\log\left(\gamma^{-1}\right)}{n}}+\sqrt{\frac{E_{\mu }\left(x^{2}\right)}{n}}. \end{array}$$

Then, with probability at least 1 − *γ*, the following holds

(A.25) $$\begin{array}{c} \left|\hat{x}-E_{\mu }\left(x\right)\right|\leq \sqrt{\frac{2E_{\mu }\left(x^{2}\right)\log\left(\gamma^{-1}\right)}{n}}+\sqrt{\frac{E_{\mu }\left(x^{2}\right)}{n}}. \end{array}$$

### B

Proof of Lemma 2

The proof process of Lemma 2 needs the next proposition [5]:

Proposition 1 . Let *σ*, *α* ∈ *ℝ* with *σ* > 0 and *α* ≥ 1. Let *μ*_1_, *μ*_2_ ∈ *ℤ*. Then,

(B.1) $$\begin{array}{c} D_{\alpha }\left(N_{\mathbb{Z}}\left(\mu_{1},\sigma^{2}\right)\left\|N_{\mathbb{Z}}\left(\mu_{2},\sigma^{2}\right)\right.\right)=\frac{{\left(\mu_{1}-\mu_{2}\right)}^{2}}{2\sigma^{2}}\alpha . \end{array}$$

Furthermore, this inequality is an equality whenever *α*(*μ*_1_ − *μ*_2_) is an integer.

Proof of Lemma 2: we can get Lemma 2 easily though Proposition 1 and Definition 3.
